# Validation of a Tablet Application for Assessing Dietary Intakes Compared with the Measured Food Intake/Food Waste Method in Military Personnel Consuming Field Rations

**DOI:** 10.3390/nu9030200

**Published:** 2017-02-27

**Authors:** Mavra Ahmed, Iva Mandic, Wendy Lou, Len Goodman, Ira Jacobs, Mary R. L’Abbé

**Affiliations:** 1 Department of Nutritional Sciences, University of Toronto, Toronto, ON M5S 3E2, Canada; mavz.ahmed@mail.utoronto.ca; 2 Faculty of Kinesiology and Physical Education, University of Toronto, Toronto, ON M5S 2W6, Canada; iva.mandic@mail.utoronto.ca (I.M.); ira.jacobs@utoronto.ca (I.J.); 3 Dalla Lana School of Public Health, University of Toronto, Toronto, ON M5T 3M2, Canada; wendy.lou@utoronto.ca; 4 Defence Research and Development Canada—Toronto Research Centre, Toronto, ON M3K 2C9, Canada; len.goodman@drdc-rddc.gc.ca

**Keywords:** dietary assessment, validation, smartphone, technology

## Abstract

The collection of accurate dietary intakes using traditional dietary assessment methods (e.g., food records) from military personnel is challenging due to the demanding physiological and psychological conditions of training or operations. In addition, these methods are burdensome, time consuming, and prone to measurement errors. Adopting smart-phone/tablet technology could overcome some of these barriers. The objective was to assess the validity of a tablet app, modified to contain detailed nutritional composition data, in comparison to a measured food intake/waste method. A sample of Canadian Armed Forces personnel, randomized to either a tablet app (*n* = 9) or a weighed food record (wFR) (*n* = 9), recorded the consumption of standard military rations for a total of 8 days. Compared to the gold standard measured food intake/waste method, the difference in mean energy intake was small (−73 kcal/day for tablet app and −108 kcal/day for wFR) (*p* > 0.05). Repeated Measures Bland-Altman plots indicated good agreement for both methods (tablet app and wFR) with the measured food intake/waste method. These findings demonstrate that the tablet app, with added nutritional composition data, is comparable to the traditional dietary assessment method (wFR) and performs satisfactorily in relation to the measured food intake/waste method to assess energy, macronutrient, and selected micronutrient intakes in a sample of military personnel.

## 1. Introduction

Dietary assessment methods have traditionally relied on tools such as 24-h recalls, food frequency questionnaires, or multi-day weighed food records (e.g., a 3 Day weighed food record) [1]. Data collection using such methods is prone to measurement errors including recall bias, respondent burden, and the researcher burden of coding recorded foods [2]. Moreover, using manual methods of collection restricts an individual’s ability to understand their food consumption patterns and nutrient intakes and limits the quick and easy analysis of dietary habits at the population level [3,4]. The current gold standard in dietary assessment methodology is the measured food intake/food waste method, wherein the amount of food not consumed is subtracted from the total amount of food given to get a precise measure of the amount consumed [5,6]. Although this method is considered more accurate than other methods that rely on an individual’s memory, the measured food intake/waste method is costly, time consuming, and onerous for both researchers and participants [5,6].

Optimizing the nutritional intake for military personnel is particularly imperative in order to meet the high-energy demands of training and operations [7]. Sufficient energy and nutrient intake promotes general health and reduces injury risk [7]. Accurately understanding the nutritional requirements to sustain the health and performance of military personnel is integral to ensuring their physiological and psychological wellbeing and operational readiness. 

Limited data exists about the quantification of energy intake in Canadian Armed Forces (CAF) personnel, who are exposed to extremely challenging training and operations, during which daily energy expenditures can be >6000 kcal/day [8,9,10,11]. Contextualizing the environment in which the participants are reporting energy and nutrient intakes is also imperative in understanding habitual dietary intakes so that the findings can be accurately extrapolated [12]. Precise methods of quantifying dietary intakes are required for both field-based researchers and health professionals (e.g., physicians, dietitians) to provide evidence-based interventions and recommendations on nutritional practices for military personnel under such conditions. However, the collection of accurate and reliable dietary intake data from military personnel is challenging in the field due to physical and cognitive stressors such as ambient temperature extremes, reduced sleep, and heavy load carriage [7,11,13,14].

Considering recent advancements and increases in adopting smart-phone technology, the use of tools such as mobile applications (apps), dietary trackers, and image capturing equipment may overcome some of the barriers associated with traditional dietary assessment methods [2] and be particularly useful for military personnel in the field. Recent reviews have indicated that the use of mobile phone technology in recording dietary intakes is preferred by participants and researchers over traditional methods, while offering the potential to reduce the burden related to coding and analysis [4,15]. 

There are several commercial mobile apps (e.g., MyFitnessPal and Lose It!) that facilitate the digital recording of dietary intakes [15,16]. Although there are studies testing the effectiveness of some of these apps in promoting health and/or weight-loss [17], limited data exist on the validation of these commercial mobile apps against the current gold standard of dietary intake methods and/or reference recovery biomarkers to assess their accuracy and reliability in assessing diets [3,18]. Carter et al. (2013) examined the validity of the use of a smartphone app and found that the app correlated with the 24-h recalls, although the limits of agreement were wide for individual energy intakes [19]. This study used a smartphone app designed for weight loss and used 24 h dietary recalls as a reference measure, which is subject to recall bias and misreporting [19]. Additionally, research assessing the use of commercial mobile apps in military personnel, especially CAF, is lacking. Although, McClung et al. (2009) have examined the monitoring of energy intake using a technology-assisted device in a military population, this study used a personal digital assistant, which is rarely used compared to smartphones [13]. Therefore, the present study aims to validate the use of the mobile app MyFitnessPal [20], which was chosen as it allows the addition of nutritional composition data for the foods provided by the study (in this case military rations or individual meal packs (IMPs)), with the current gold standard in dietary assessment methodology; measured food intake with weighed food waste. The results were also compared with reference recovery biomarker data.

## 2. Materials and Methods 

### 2.1. Study Participants

This research was conducted at Defence Research and Development Canada (DRDC), Toronto, Ontario between January 2014 and May 2015. The participants were 18 CAF (mean age 34 years) personnel who were Regular Force or Class A Reservists participating in a concurrent laboratory metabolism and feeding study. A detailed description of the study can be found in the technical report prepared by University of Toronto for DRDC [10]. A total of 27 participants initially volunteered for the study. Two participants never started the protocol, and an additional seven participants dropped out due to scheduling difficulties and/or non-compliance due to the demanding nature of the protocol. All participants provided written informed consent to participate in the study. The Research Ethics Boards at both DRDC (approval code 2013-075) and the University of Toronto (approval code 29914) approved the study.

### 2.2. Demographic and Anthropometric Assessments

Participants were asked to complete a questionnaire on demographics. The anthropometric measurements included height, weight, and body fat percentage. Body weight and height were measured without shoes, with light clothing and using standard calibrated equipment (height and weight scales). Body composition (including percent body fat) was assessed using air-displacement plethysmography (BOD POD™: This is a machine that measures body composition via densitometry). Body mass index (BMI) was calculated as the body weight (kg) divided by the height (m) squared. 

### 2.3. Study Procedures

Upon written informed consent and completion of the pre-study questionnaires, participants were randomized to either the wFR or the tablet app (full details below). Out of 18 menu items (6 breakfast, 6 lunch, and 6 dinner), participants selected three standard CAF ration packs (IMPs)/day for two consecutive days each week for four weeks. The rations contain pre-packaged pre-labelled food and beverage items (e.g., sliced apples, bread, coffee, breakfast sausages, etc.). Although, participants could only consume the beverages (sports drink, coffee, tea, vanilla cappuccino) provided within the rations, they were able to have water ad libitum. Participants were asked to record their consumption of rations using either the wFR or the tablet app for the duration of the study. All participants were trained in documenting, weighing, and measuring their dietary intake using both methods and were provided with written instructions for reference during the recording period. On the third day, participants were instructed to bring back all food waste from the unconsumed and/or partially consumed ration packs/IMPs, which served as the reference method for the evaluation of each of the two test methods. Study investigators (Mavra Ahmed (MA) and Iva Mandic (IM)) reviewed the food record or tablet app details with each participant for each two-day recording period in each of the four weeks. This review involved the clarification of items that may have had missing quantities or may have been misspelled or illegible in the case of the food record. 

At baseline and at the end of the study, the participants were asked to complete a brief questionnaire on their knowledge, attitude, and behaviour regarding prior or current use of mobile technology and the perceived usefulness and ease of using the tablet app. Response options to the questions included both open ended text as well as Likert-scaled responses on a scale of 1–5. 

The dietary intakes of participants were derived from food and beverages only for a total of two days each week for four weeks for each method; wFR (*n* = 9) and the tablet app (*n* = 9). Participants were asked to refrain from the consumption of foods other than the rations. However, in the case where participants were to consume other foods, they were asked to report the intake accordingly. We only had one instance in which a participant had consumed a bowl of salad on one day out of 8 days. We clarified the contents with the participant and ran the analysis with it included. Participants were asked to refrain from vitamin and mineral supplements for the duration of the study. The nutrient values for the combat rations or IMPs were provided by the CAF Directorate of Food Services. For nutrients that were missing (B-vitamins, potassium, magnesium, phosphorus, zinc), values were taken from similar foods in the Canadian Nutrient File 2013 as part of the ESHA© (Elizabeth Stewart Hands and Associates) Food Processor Nutrition Analysis, version 10.13.1, 2013, ESHA Research, Salem, OR, USA) database. 

Errors made by the participants (e.g., participants may have over- and/or under-reported some items as well as omitting a recording of some items) and outliers were not removed in order to provide an accurate indication of the relative validity of the different dietary assessment methods.

Food records (either reported using the wFR or the tablet app) were entered by two trained coders using a nutrient software program (ESHA© Food Processor Nutrition Analysis, version 10.13.1, 2013, ESHA Research, Salem, OR, USA) and double-checked and analyzed by a trained study investigator (MA). 

### 2.4. Weighed Food Record (wFR)

Participants using the wFR method recorded the time, place, and a detailed description about each consumed food and beverage item. Participants were provided with household measuring utensils and a standard food scale (PrepTech, PT-800, Newport Beach, CA, USA) to weigh each food item. 

### 2.5. Tablet App

Participants using the tablet app, MyFitnessPal, were provided with a Samsung^®^ Galaxy Tab 3/Note 3 with the app pre-downloaded. The app, MyFitnessPal, had a full list of CAF rations/IMPs nutritional information added to the database by a study investigator (MA). Participants were able to search for their food/beverage item of choice and add it to the respective meal; breakfast, lunch, dinner, and/or snacks. All items within the combat rations/IMPs were packaged in pre-determined quantities. Participants were provided with household measuring utensils and a standard food scale (PrepTech, PT-800, Newport Beach, CA, USA) to weigh each food item.

### 2.6. Measured Food Intake/Weighed Food Waste Method (Reference Method)

Participants selected three ration packs per day and were asked to bring back all unconsumed and/or partially consumed food and beverage items. The study investigators (MA and IM) weighed and recorded all the partially consumed food and beverage items to the nearest gram or millilitre, using a standard food scale (PrepTech, PT-800, Newport Beach, CA, USA). The measured food intake/food waste was calculated from the amount unconsumed subtracted from the known quantity of each menu item selected and brought home.

### 2.7. Urinary and Blood Biomarkers

Participants were instructed to collect their urine for 24 h immediately before coming back to DRDC for fasting blood collection on Day 3 of each week. Participants were provided with a plastic container to collect their urine and a leak-proof bag in which to store their urine container. Participants were instructed to discard the first urine sample of the day and to collect all subsequent urine for the next 24 h, including the first urine sample on the following day. 

Venous blood samples (10 mL) were collected from each participant after an overnight fast. Erythrocytes and plasma were separated within 1 h of collection. Both the urine and plasma samples were shipped to a third party blood chemistry laboratory (Lifelabs, Toronto, ON, Canada) for processing. 

Creatinine excretion was used to assess the adequacy of the urine collections by using creatinine excretion standards (<8.8 mmol/day for males and <4.5 mmol/day for females) [21].

### 2.8. Statistical Analysis

All data are presented as mean ± Standard Deviations (SD). Multiple regression for repeated measurements was used to examine the relationship between the nutrient intake data estimated using the tablet app or wFR (dependent variables) and the measured food intake/waste method (predictor variable), adjusted for multiple days [22]. The Repeated Measures Linear Mixed Model was used to test for differences in the data collected using the tablet app or the wFR and the measured food intake/waste method, adjusted for 8 days of recording and collection per method. A Repeated Measures Bland-Altman [23,24] analysis was used to assess the relative bias (mean difference) and random error (1.96 Standard Deviation (SD) of the difference) between the tablet app or the wFR with the measured food intake/waste method. Correlations for association between dietary intakes (averaged for two consecutive days over four weeks) (dependent variable) and urinary/blood biomarkers (four collections per participant) (predictor variables) with adjustments for age, energy intake, and body mass index as possible covariates were obtained using multiple regression [22]. All data were analysed using SPSS Statistics (version 24, 2016; IBM Corporation^®^, Armonk, NY, USA), and statistical significance was set at *p* ≤ 0.05. 

## 3. Results

### 3.1. Participant Demographics and Anthropometrics

The participants’ demographics and anthropometric measurements are summarized in Table 1. Of the 18 CAF participants who participated in the study, 78% were male and 67% were Caucasians. The mean age of participants was 34 ± 11 years, with a mean BMI of 26 ± 3.6 kg/m^2^ and a mean percent body fat of 23% ± 8.1%. The majority of the participants had a university degree (61%).

### 3.2. Smartphone and Tablet Usage

A vast majority (89%) of our participants used a smartphone as their primary phone on a daily basis, and 67% used it to keep track of their physical activity and dietary habits. Of the tablet users (*n* = 9), 89% of users found the tablet app easy to use, 67% found it comfortable to carry around, and 56% thought the app helped them to record their food items accurately. 

### 3.3. Comparison of Tablet App with Measured Food Intake/Food Waste Method

There were highly significant correlations between the tablet app and the measured food intake/waste method for both macro- and micro-nutrients (correlations ranging from 0.963 to 0.999; *p* ≤ 0.05) (Table 2). The differences between the methods were not significantly different (*p* > 0.05) for nutrients energy, carbohydrates, fat, saturated fat, protein, vitamin A, vitamin C, calcium, iron, and sodium. For all of these nutrients, the tablet app yielded lower intakes than the measured food intake/waste method; with intakes approximately 3% lower for energy and carbohydrates, 4% lower for fat, 2% lower for protein, and 3%–12% lower for micronutrients. 

For energy intake, the mean difference between the tablet app and the measured food intake/waste method was not significant (−73 kcal/day; 95% Confidence Interval (CI) for bias = −109 to −37 kcal/day) (*p* > 0.05). Although the tablet app had a 3% bias towards under-reporting energy intake in comparison to the measured food intake/ waste method, the 95% CI for this bias was narrow (−1.5% to −4.5%). For random error, the 95% lower and upper Limits Of Agreement (LOA) between the methods for energy intake ranged from −250 to 104 kcal/day. 

Similarly, the mean difference for carbohydrate (−12 g/day), fat (−2.21 g/day) and protein intakes (−1.25 g/day) was not significant (*p* > 0.05), with a narrow 95% LOA (−43 to 19 g/day for carbohydrates, −9 and 4.5 g/day for fat and −5.5 to 3 g/day for protein). Similar results were found for other nutrients (Table 2). 

**Figure 1:**
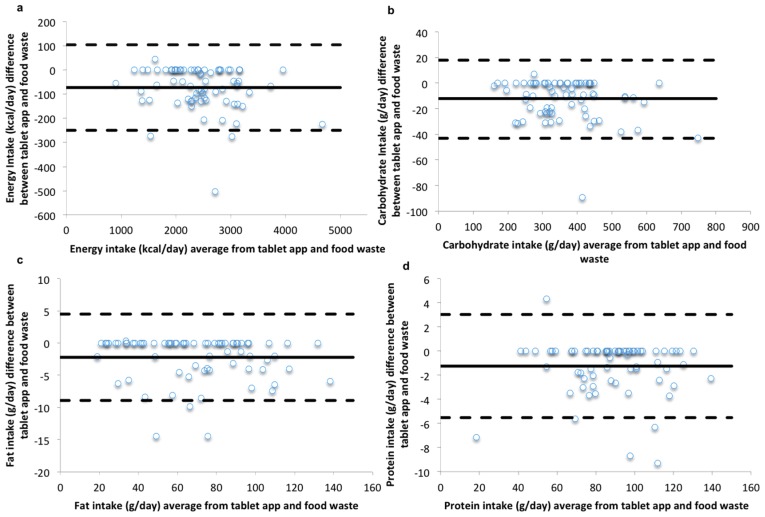
Repeated Measures Bland-Altman plots of the difference between intakes recorded by the tablet app and those from the measured food intake/food waste method against the mean values for the two methods for (**a**) energy; (**b**) carbohydrates; (**c**) fat; and (**d**) protein. The solid line indicates the mean difference (energy −73 kcal/day, carbohydrates −12 g/day, fat −2.21 g/day and protein −1.25 g/day), and the dashed line indicates the 95% Limits Of Agreement (LOA) (1.96 SD) for nutrient intakes (energy −250 kcal/day, 104 kcal; carbohydrates −43 g/day, 18 g/day; fat −8.91 g/day, 4.5 g/day and protein −5.52 g/day, 3.03 g/day).

The Bland-Altman plots (Figure 1) for energy, macronutrient, and micronutrient intakes demonstrate that data for most participants were within the LOA with few outliers. There was no apparent proportional bias, suggesting that the differences between the two methods occurred at random across the range of intakes. 

### 3.4. Comparison of Weighed Food Record (wFR) with Measured Food Intake/Waste Method

There were highly significant correlations between the wFR and the measured dietary intake for both macro- and micro-nutrients (correlations ranging from 0.904 to 0.996, *p* < 0.001), and there were no differences between nutrients (*p* ≥ 0.05) (Table 3). For all of the nutrients, the participants reported lower intakes using the wFR than that obtained from the measured dietary intake, with intakes approximately 3.5% lower for energy and fat, 4% lower for carbohydrates, 2% lower for protein, and 0% to 19% lower for micronutrients (Table 3). 

For energy intake, the mean difference between the wFR and the measured dietary intake was small (−108 kcal/day, *p* ≥ 0.05), with a 95% lower and upper LOA of −338 to 122 kcal/day, respectively. Similar to the tablet app, the wFR method had a 3.5% bias towards underreporting, although the 95% CI for bias was narrow (5% to 2%). The mean differences for carbohydrate (−19 g/day), fat (−3 g/day), and protein (−1.8 g/day) intakes were small (*p* > 0.05) with a narrow 95% LOA (−58 to 19 g/day for carbohydrates, −11 and 5 g/day for fat and −8 to 4 g/day for protein). Similar results were found for other nutrients, where no mean difference (*p* > 0.05) and a narrow 95% LOA between the methods (Table 3) was seen. 

**Figure 2:**
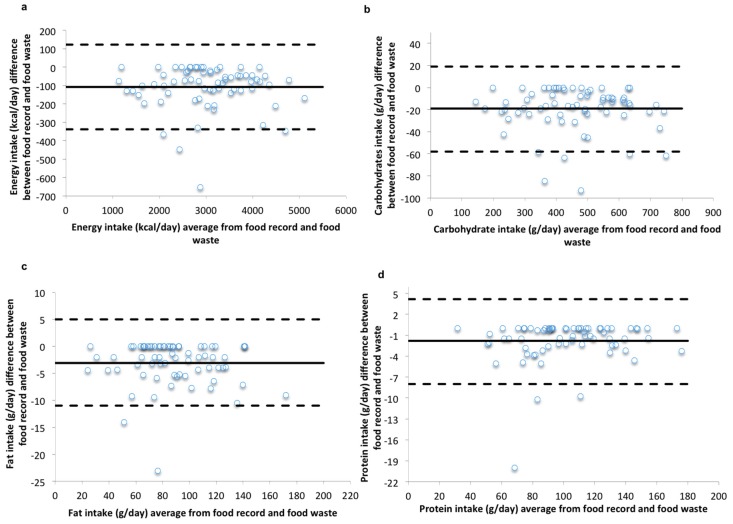
Repeated Measures Bland-Altman plots of the difference between intakes recorded by the wFR and those obtained from the measured food intake/waste method against the mean values for the two methods for (**a**) energy; (**b**) carbohydrates; (**c**) fat; and (**d**) protein. The solid line indicates the mean difference (energy −108 kcal/day, carbohydrates −19 g/day, fat −3.1 g/day and protein −1.8 g/day) and the dashed line indicates 95% LOA (1.96 SD) for nutrient intakes (energy −338 kcal/day, 122 kcal; carbohydrates −58 g/day, 19 g/day; fat −11 g/day, 5 g/day, and protein −8 g/day, 4.2 g/day).

The Bland-Altman plots (Figure 2) for energy and macronutrient intakes demonstrate that most participants fell within the LOA with few outliers in the data. There was also no apparent proportional bias, suggesting that the differences between the two methods occurred at random across the range of intakes. 

### 3.5. Relationships between Dietary Assessment Methods and Biomarkers of Intake

**Figure 3:**
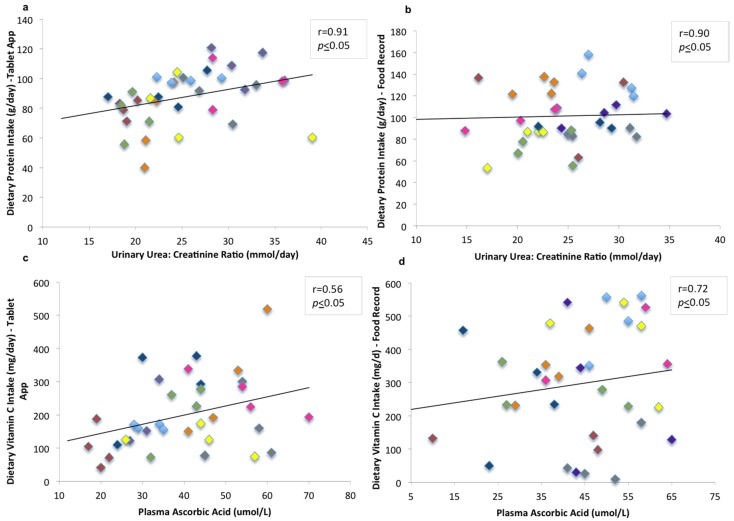
Scatter plots of the relationship between (**a**,**c**) urinary urea:creatinine ratio and dietary protein intake and between (**b**,**d**) plasma ascorbic acid and dietary vitamin C intake recorded by the (**a**,**b**) tablet app and by (**c**,**d**) wFR. The correlations are examined using multiple regression for repeated measurements and are adjusted for age, body mass index, and energy intake. Data presented is for dietary intake (two days averaged per week for four weeks = four days) and urinary and blood biomarkers (four collections) per participant. The four sets of colors represent individual participants.

The data showed moderate to good correlations where the urinary urea:creatinine ratio (unadjusted, *r* = 0.34; adjusted *r* = 0.91) and the plasma ascorbic acid (unadjusted, *r* = 0.35; adjusted *r* = 0.56) were significantly related to dietary intakes using the tablet app (*p* ≤ 0.05) (Figure 3a,b) after the adjusted values were controlled for age, energy intake, and body mass index.

For the relationship between dietary intakes reported using the wFR and the biomarkers of intake, urinary urea:creatinine ratio (unadjusted, *r* = 0.21; adjusted *r* = 0.90) was significantly related to protein intake (*p* ≤ 0.05) after adjustment. Without adjustment, ascorbic acid was not significantly related to vitamin C intake (*r* = 0.2; *p* > 0.05), but when controlled for age, body mass index, and energy intake, the correlation coefficient between vitamin C intake and plasma ascorbic acid was significantly positively correlated (*r* = 0.72, *p* ≤ 0.05) (Figure 3c,d).

## 4. Discussion

The present study demonstrated that a tablet app with integrated military ration nutritional information data is comparable to the traditional wFR method to assess dietary intakes in military personnel under non-operational settings. 

Our study showed that the participants preferred to use the tablet app to keep track of their dietary intake (in contrast to the food record when they had the opportunity to try both methods prior to the study), which was in agreement with Jospe et al. [3], who found a positive perception of diet app usage by dietitians to assess or track intakes. In addition, Lieffers et al. [25] also indicated that diet apps were convenient and easy to use for keeping track of dietary intake.

This study demonstrated good correlations between using the tablet app with the measured food intake/waste method for total energy, macronutrient, and micronutrient intakes. These correlations compared favourably with results from other validation studies of technology-assisted dietary assessment methodologies [13,15,19,26,27]. Carter et al. [19] compared a smartphone app with 24-h recalls and found correlations ranging from 0.63 to 0.83, which are quite a bit lower than those seen in our study. Our findings of high correlations between the tablet app and the measured food intake/waste method are likely due to the use of standardized pre-weighed rations with known nutrition information. Similarly, our findings demonstrated good correlations between the wFR and the measured food intake/waste method, indicating that measurement of nutrient intake levels using the tablet app is comparable to that from the wFR; which is currently considered a robust method of assessing diets when information on multiple days of dietary intake has been recorded [28].

The Bland-Altman plots indicated a good level of agreement between each method (tablet app or wFR) and the measured food intake/waste method at a range of intakes, with most of the data points located within the 1.96 SD of the mean (narrow LOA). This indicates that the tablet app is suitable for accurately estimating intakes at an individual level. Our finding of narrow LOA is consistent with the results by Timon et al. [26] but in contrast to studies comparing either a smartphone app or a Personal Digital Assistant (PDA) with 24-h recall [15,19,29]. As suggested by the authors of these latter studies, this could be possibly due to measurement errors found in the reference measure (24-h recall), which itself is not a measure of absolute intakes as it is not representative of habitual intakes and is prone to recall bias and misreporting [19], whereas we were able to measure absolute food intake with our use of the current gold standard (measured food intake/food waste method).

Although the study showed relatively good agreement between the two methods, as illustrated by the Bland-Altman analysis, there were lower mean daily dietary intakes reported by the tablet app than by the measured food intake/waste method (although these were not significantly different), reflecting some measure of systematic bias. Similar results were found for the agreement between the wFR and the measured food intake/waste method. However, it should be noted that the participants in this study were consuming standardized military rations with low variability in their nutrient compositions, and the variety of food items from which the choices were made was quite narrow, therefore further reducing variation between participants and the potential to underreport. In light of the relatively narrow variety in menu items available in rations, we used the repeated measures linear mixed model approach, which utilizes the repeated measurements on each individual while accounting for variation within participants. Additionally, the magnitude of the bias in energy intakes from both the tablet app (−73 kcal/day) and the wFR (−108 kcal/day) were small with a narrow 95% CI for bias. Our findings of underreporting in both the methods are lower than the energy intake of 110 to 165 kcal/day, which is considered to be clinically meaningful for weight loss [12,30]. This suggests that the under-reporting seen in this study will not likely impact energy balance in this sample of CAF personnel and that the tablet app could be a valuable tool for self-reporting dietary intakes in a sample of military personnel under non-operational conditions. 

Although limited studies exist in quantifying the accuracy of energy intakes within military personnel using mobile phone technology, our findings of underreporting of 3% and 3.5% are lower than those seen in national population-based surveys, in which the underreporting of energy intake has been estimated to range from 10% to 20% [31,32]. The low rate of underreporting in our study is also an improvement in comparison to some studies, using the food record and/or 24-h recalls as a reference method, that underestimate energy intake by 6% or more [33,34,35]. Similar to our findings, some studies using training/reminders, weighed meals, and/or total energy expenditure as a reference method in evaluating or validating digital-assisted dietary assessment methods have found underreporting ranging from 3% to 4% [13,36]. Participants in our study were shown and given written instructions on how to weigh and measure food quantities and how to use both the wFR and the tablet app methods to record dietary intakes. Additionally, military personnel have been shown to display traits of higher inherent motivation levels, especially when it comes to enhancing performance and maintaining weight [37]. All of these factors may have contributed to improved compliance among our participants, which may explain the low bias and good agreement demonstrated in this study. Thus, the small underreporting is likely a true reflection of a systematic bias of 3%–4% using these methods (tablet app or wFR). 

There are several possible reasons for the finding of small under-reporting from the tablet app evident in this study; the most important being that participants were able to see the feedback display of nutrient intakes on the app [18], which may have resulted in an unintended behavioural change. Alternatively, participants may have failed to enter all of the food items and may not have provided accurate assessments of food portion sizes [19,26,27,38], although the latter is unlikely because the ration packages are all pre-weighed and participants were provided with food scales and measuring cups/spoons to weigh the leftovers. Finally, the small underreporting seen in both the tablet app and wFR is possibly due to the burden upon respondents to record detailed dietary information by pen/pencil or by typing [4]. 

Reference biomarkers of dietary intake provide an objective method to validate selected components of dietary intakes [26,39]. Using multiple regression and after adjusting for covariates, we found a positive significant correlation between the dietary intakes from both the wFR and the tablet app with the biomarkers. Our findings are similar with the results found in another study validating technology-assisted dietary assessment methods with biomarkers [26]. Some of the lack of association for the unadjusted correlation is probably due to our small sample size, differential misreporting, or covariates such as energy intake, BMI, and age, which, when added to the data, improved the correlations. We also noticed that individuals using the tablet app recorded a 5% lower intake of sports drinks (which contain high amounts of vitamin C) in contrast to participants using the wFR, which may explain the lower association between the reported vitamin C intake using the tablet app and plasma ascorbic acid. One of the main strengths of our study is the validation of a tablet app in assessing dietary intakes in a sample of military personnel consuming food/beverages of known nutritional composition with the food waste method, the current gold standard in assessing diets [28,40]) and biomarkers in a real world setting (at home over a period of 8 days). However, it is important to acknowledge that the majority of the participants in our study were highly compliant and appeared to be comfortable with the use of technologies; therefore, the results of our study may not be generalizable to the general healthy Canadian adult population. In addition, our study investigators conducted one-on-one training for each participant to ensure accurate recording of dietary intake, which may not be feasible for larger studies. 

## 5. Conclusions 

Current findings suggest that a tablet app, when modified to contain detailed nutritional composition data, is comparable to the traditional method of assessing dietary intakes (wFR). The tablet app also performed satisfactorily compared to the measured food intake/food waste method (current gold standard) and could offer a mobile alternative to the wFR for the estimation of dietary intake in a sample of CAF personnel under operational conditions. 

Although promising as an alternate dietary assessment method for monitoring the dietary intake of military personnel, the tablet app still needs to be validated in a larger sample size and under military operational settings of added physical and psychological stress.

## Figures and Tables

**Table 1:** Characteristics (demographics and anthropometrics) of the study participants.

Characteristics	*n* = 18
Age (year) ^1^	34 ± 11
Sex ^2^	
Male	14 (78%)
Female	4 (22%)
Height (cm) ^1^	174 + 10
Weight (kg) ^1^	79 + 13
Body Mass Index (kg/m^2^) ^1^	26 ± 3.6
Percent Body Fat (%) ^1^	23 ± 8.1
Ethnicity ^2^	
Caucasian	12 (67%)
Asian	3 (17%)
African American	1 (6%)
Hispanic	1 (6%)
Other	1 (6%)
High-school graduation	1 (6%)
Non-university certificate	6 (33%)
University Degree	11 (61%)
Marital Status ^2^	
Single	12 (67%)
Married	5 (28%)
Separated	1 (6%)

^1^ Mean ± Standard Deviations (SD). ^2^ *n* (%).

**Table 2:** Daily energy and nutrient intakes recorded by Canadian Armed Forces (CAF) personnel participants using the tablet app ^1^ (*n* = 9) compared to the measured food intake/food waste method ^2^ (reference method) (Means ± Standard Deviations (SD)).

Nutrients ^†^	Tablet App ^1^ *n* = 9	Measured Food Intake/Waste Method ^2^	Correlation Coefficient (*r*)	Difference	Limits of Agreement (LOA) ^§^		*p*-Value **
	Mean (SD)	Mean (SD)		Mean (SD)	Lower	Upper	
Energy (kcal/day)	2410 (651)	2484 (670)	0.992 *	−73 (89)	−250	104	0.42
Carbohydrates (g/day)	359 (110)	371 (113)	0.992 *	−12 (16)	−43	18	0.47
Fat (g/day)	68 (29)	71 (29)	0.993 *	−2 (3.4)	−9	4.5	0.46
Saturated Fat (g/day)	24 (11)	25 (11)	0.993 *	−0.8 (1)	−3.4	1.8	0.53
Protein (g/day)	87 (23)	89 (23)	0.996 *	−1.3 (2)	−5.5	3	0.71
Vitamin A (μg/day)	7 (41)	8 (41)	0.999 *	−0.3 (1.5)	−3.4	2.7	0.96
Vitamin C (mg/day)	200 (160)	211 (160)	0.985 *	−11 (28)	−67	45	0.66
Calcium (mg/day)	513 (223)	532 (220)	0.989 *	−19 (35)	−88	50	0.49
Iron (mg/day)	18 (6)	19 (6)	0.994 *	−0.4 (0.7)	−2	1	0.64
Sodium (mg/day)	3725 (1061)	3835 (1083)	0.963 *	−109 (292)	−684	465	0.44

* *p* ≤ 0.05; ** *p*-value is the significance level for differences between two methods. ^§^ Lower and upper Limits Of Agreement (LOA) (mean difference ± 1.96 SD). ^†^ Energy and nutrient intake data examined by Multiple Regression and differences estimated by Repeated Measures Linear Mixed Models. ^1^ Tablet App; tablet was preloaded with MyFitnessPal app software, which was modified to contain nutritional composition of all possible military ration choices. ^2^ Measured Food Intake/Waste Method; all consumed and/or non-/partially consumed food and beverage items from the military ration packs were weighed and recorded for each participant.

**Table 3:** Daily energy and nutrient intakes recorded by CAF personnel using the weighed food record (wFR) ^1^ and those obtained from the measured food intake/food waste method ^2^ (reference method) (Means ± Standard Deviations (SD)).

Nutrients ^†^	Weighed Food Record ^1^ *n* = 9	Measured Food Intake/Waste Method ^2^	Correlation Coefficient (*r*)	Difference	Limits of Agreement (LOA) ^§^		*p*-Value **
	Mean (SD)	Mean (SD)		Mean (SD)	Lower	Upper	
Energy (kcal/day)	2972 (900)	3080 (902)	0.993 *	−108 (117)	−338	122	0.32
Carbohydrates (g/day)	449 (145)	469 (146)	0.993 *	−19 (20)	−58	19	0.30
Fat(g/day)	87 (30)	90 (30)	0.992 *	−3.1 (4)	−11	5	0.38
Saturated Fat (g/day)	32 (11)	33 (12)	0.993 *	−0.96 (1.4)	−4	2	0.51
Protein (g/day)	101 (31)	103 (30)	0.996 *	−1.8 (3)	−8	4.2	0.65
Vitamin A (μg/day)	7 (8)	9 (8)	0.904 *	−1.8 (3.7)	−9.1	5.5	0.09
Vitamin C (mg/day)	300 (201)	307 (201)	0.994 *	−6.7 (23)	−52	39	0.80
Calcium (mg/day)	619 (297)	655 (297)	0.984 *	−36 (59)	−152	79	0.37
Iron (mg/day)	22 (9)	22 (9)	0.995 *	−0.6 (0.96)	−2.5	1.3	0.60
Sodium (mg/day)	4640 (1228)	4759 (1256)	0.987 *	−119 (212)	−535	297	0.48

* *p* ≤ 0.05; ** *p*-value is the significance level for differences between two methods. ^§^ Lower and upper limits of agreement (LOA) (mean difference ± 1.96 SD). ^†^ Energy and nutrient intake data examined by Multiple Regression and differences estimated by Repeated Measures Linear Mixed Models. ^1^ Weighed Food Record (wFR); participants using the wFR method were asked to weigh and record each consumed food and beverage item on a paper-based self-report form. ^2^ Measured Food Intake/Waste Method; all consumed and/or non-/partially consumed food and beverage items from the military ration packs were weighed and recorded for each participant.
